# ERK1b, a 46‐kDa ERK isoform that is differentially regulated by MEK

**DOI:** 10.1002/cbin.11801

**Published:** 2022-04-04

**Authors:** Yuval Yung, Zhong Yao, Tamar Hanoch, Rony Seger

**Affiliations:** ^1^ Department of Biological Regulation The Weizmann Institute of Science Rehovot Israel

**Keywords:** alternative splicing, ERK, MAPK, nuclear localization, phosphorylation

## Abstract

The extracellular signal‐regulated kinases (ERK) 1 and 2 (ERK1/2) are members of the mitogen‐activated protein kinase family. Using various stimulated rodent cells and kinase activation techniques, we identified a 46‐kDa ERK. The kinetics of activation of this ERK isoform was similar to that of ERK1 and ERK2 under most but not all circumstances. We purified this isoform from rat cells followed by its cloning. The sequence of this isoform revealed that it is an alternatively spliced version of the 44‐kDa ERK1 and therefore we termed it ERK1b. Interestingly, this isoform had a 26‐amino acid insertion between residues 340 and 341 of ERK1, which results from Intron 7 insertion to the sequence. Examining the expression pattern, we found that ERK1b is detected mainly in rat and particularly in Ras‐transformed Rat1 cells. In this cell line, ERK1b was more sensitive to extracellular stimulation than ERK1 and ERK2. Moreover, unlike ERK1 and ERK2, ERK1b had a very low binding affinity to MEK1. This low interaction led to nuclear localization of this isoform when expressed together with MEK1 under conditions in which ERK1 and ERK2 are retained in the cytoplasm. In addition, ERK1b was not coimmunoprecipitated with MEK1. We identified a new, 46‐kDa ERK alternatively spliced isoform. Our results indicate that this isoform is the major one to respond to exogenous stimulation in Ras‐transformed cells, probably due to its differential regulation by MAPK/ERK kinase and by phosphatases.

## 1 INTRODUCTION

The mitogen‐activated protein kinase (MAPK) cascades play a central role in transmitting the signals of many extracellular stimuli. The four MAPK cascades (extracellular signal‐regulated kinase [ERK], p38, c‐Jun N‐terminal kinase [JNK], and ERK5) known today consist of a core of three protein kinases that sequentially phosphorylate and activate the downstream components (Flores et al., 2019; Lee et al., 2020; Plotnikov et al., 2011). In some cells or stimuli, the cascades may contain additional layers of protein kinases that contribute to signaling versatility. In most cases, these cascades transmit signals from receptors in the plasma membrane to various intracellular targets, either in the cytoplasm or the nucleus. These signals regulate a variety cellular processes including proliferation, differentiation, stress responses, and others. The ERK cascade that has two main isoforms in its MAPK tier (ERK1/2; Eblen, 2018; Klomp et al., 2021; Lavoie et al., 2020; Maik‐Rachline et al., 2019) was the first MAPK cascade identified. This cascade was shown to participate mainly in the induction and regulation of proliferation and differentiation. Upon growth factor stimulation, the signals through the cascade is initiated by activated Ras. This small GTPase, in turn, transmits the signal to the first protein kinase in the cascade, which is Raf. Then the signal proceeds by phosphorylation and activation of MAPK/ERK kinase (MEK), ERK, and several MAPK activated protein kinases (MAPKAPKs) like p90 ribosomal S6 kinase (RSK). Importantly, the ERK cascade plays a role in other processes such as morphology determination (Maik‐Rachline et al., 2019; Simon et al., 2020) and in learning and memory processes in the brain (Miningou & Blackwell, 2020).

ERK is known to have two isoforms, the 44‐kDa ERK1 and the 42‐kDa ERK2, which have a high degree of homology between them (Boulton, Nye, et al., 1991). Both these isoforms are inactive in quiescent cells, but are rapidly activated upon stimulation. The activation of ERK requires phosphorylation of threonine and tyrosine residues within a TEY motif of their activation loop (Canagarajah et al., 1997; Payne et al., 1991). This activating phosphorylation is mediated by MEKs (MEK1/2; Ahn et al., 1991; Seger, Seger, et al., 1992), which are the sole activators of ERK, phosphorylating both regulatory threonine and tyrosine (Seger, Ahn, et al., 1992). However, ERK activity is regulated also by the action of various phosphatases that remove the phosphates from each of the regulatory residues, which leads to ERK inactivation (Caunt and Keyse, 2013; Yao & Seger, 2004). In the past few years, it became clear that ERK1 and ERK2 are important signaling proteins that phosphorylate regulatory substrates either in the cytosol (e.g., phospholipase a [PLA2]; Lin et al., 1993) or in the nucleus (e.g., Elk1; Chen et al., 1992; Marais et al., 1993). In addition, ERK can transmit the signal to MAPKAPK that extends the length of the cascade. The main MAPKAPK downstream of ERK is RSK (Roux & Topisirovic, 2018), which phosphorylates and activates other of substrates, leading to a large number of ERK‐dependent phosphorylated proteins. Other MAPKAPKs of the ERK cascade are MAP Kinase‐interacting kinases and mitogen‐ and stress‐activated protein kinase, but these enzymes are also activated by p38 (Cargnello & Roux, 2011).

Additional mode of ERK regulation is its dynamic subcellular localization. In resting cells, ERK is localized primarily in the cytosol, where it is retained by various anchoring proteins, including cytoskeletal elements (Wainstein & Seger, 2016). Interestingly, extracellular stimulation causes a rapid change in this localization, leading to translocation of ERK and RSK into the nucleus (Chen et al., 1992). These kinases do not contain the canonical nuclear localization signal and the mechanism of nuclear ERK translocation was shown to be mediated by a specific nuclear translocation signal that binds to the karyopherin Importin7 (Imp7; Chuderland et al., 2008). One of the proteins that retain ERK in the cytoplasm of resting cells is MEK (Fukuda et al., 1996; Rubinfeld et al., 1999). This retention is mediated by interaction of the N‐terminal domain of MEK and a nine amino acid stretch in an area close to the C terminus of ERK (Plotnikov et al., 2015, 2019). Upon stimulation, the phosphorylation of the TEY domain induces a conformational change that causes detachment of ERK from MEK or the other cytosolic anchor, allowing the free ERK to interact with Imp7 and translocate into the nucleus.

Monoclonal antibody (Ab) that specifically recognize the phosphorylated form of ERK that we have developed (doubly phosphorylated ERK [DP‐ERK]; Yung et al., 1997) are a very good tool to detect ERK1 and ERK2 phosphorylation and activity. Here we used this Ab to study ERK in rat cell lines. Interestingly, aside of the expected 42‐ and 44‐kDa ERKs, the DP‐ERK Ab detected an additional band with a molecular mass of 46 kDa. This band may be related to a 46‐kDa band that was previously recognized by anti‐C‐terminal domain of ERK Ab and tentatively termed ERK4 (Boulton & Cobb, 1991). Here we cloned this 46‐kDa ERK and found that it is an alternatively spliced form of ERK1, which we termed ERK1b. The ERK1b is abundant mainly in rat tissues, while essentially no expression was found in most other organisms, including human. We showed that ERK1b is readily activated by MEK, but the kinetic of this activation was not identical to that of ERK1. This is probably due to different mode of ERK1b regulation by phosphatases and localization. The different mode of regulation was apparent primarily in Ras‐transformed Rat1 cells, where stimulation induced distinct activation of ERK1b as compared with the other ERKs. These results strongly suggest that ERK1b plays an important role in transmitting signals when ERK1 and ERK2 are under tight downregulation by phosphatases or other enzymes.

## 2 MATERIAL AND METHODS

### 2.1 Buffers

Buffer A contains 50 mM β‐glycerophosphate, pH 7.3, 1.5 mM EGTA, 1 mM EDTA, 1 mM dithiothreitol, and 0.1 mM sodium vanadate. Buffer H (homogenization buffer) contains 50 mM β‐glycerophosphate, pH 7.3, 1.5 mM EGTA, 1 mM EDTA, 1 mM dithiothreitol, 0.1 mM sodium vanadate, 1 mM benzamidine, 10 μg/ml aprotinin, 10 μg/ml leupeptin, and 2 µg/ml pepstatin A. Buffer R (reaction mixture at threefold final concentration) contains 30 mM MgCl_2_, 4.5 mM dithiothreitol, 75 mM β‐glycerophosphate, pH 7.3, 0.15 mM sodium vanadate, 3.75 mM EGTA, 30 μM calmidazolium, and 2.5 mg/ml bovine serum albumin.

### 2.2 Preparation of cell extracts and western blot analysis

Cells were grown to subconfluence and were then serum‐starved for 18 h in Dulbecco's modified Eagle's medium containing 0.1% fetal calf serum. The cells were then exposed to various stimuli for variable amount of time. Then, the medium was removed and the cells were rinsed twice with ice‐cold phosphate‐buffered saline (PBS) and once with ice‐cold Buffer H. Cells were scraped into Buffer H (0.5 ml/plate) and disrupted by sonication (2 pulses for 7 s of 50 W) on ice. The extracts were centrifuged (100,000*g*, 15 min, 4°C) and the supernatants, which contained the cytosolic and nuclear proteins, were further kept at 4°C. The supernatants were then resolved by a 10% sodium dodecyl sulfate–polyacrylamide gel electrophoresis (SDS‐PAGE), transferred onto a nitrocellulose membrane, and probed with appropriate Ab. Abs binding was detected using alkaline phosphatase (Promega) or ECL (Amersham Pharmacia Biotech) according to the manufacturer's instructions. The Abs used were anti‐C terminus of ERK‐Ab (C‐16, Santa‐Cruz Biotechnology), anti‐hemagglutinin (HA)‐Ab (Santa Cruz Biotechnology), DP‐ERK (Sigma), and Ab 4086 (see below).

### 2.3 Generation of a polyclonal anti‐ERK1b Ab (Ab 4086)

The Ab was raised against the part of the ERK1b‐specific peptide (VSRPPAAGRGISVPSVRPVPYC) by the Ab Unit of The Weizmann Institute of Science. For immunization of rabbits, the peptide was conjugated to keyhole limpet hemocyanin using the Imject Maleimide Activated kit (Pierce).

### 2.4 Northern blot analysis

Northern blotting was performed on a Human Multiple Tissues Northern blot (Clontech). The probe used for detection was the ERK1b‐specific 78 bp insert prelabeled with [α‐^32^P]dCTP.

### 2.5 Polymerase chain reaction (PCR) and reverse‐transcriptase PCR (RT‐PCR)

PCR was made with Taq (Promega) and RT‐PCR with RT‐PCR Beads (Amersham Pharmacia Biotech) according to the manufacturer's instructions with the oligonucleotides as follows: ERK1‐NT: 5′‐STGGTGAAGGGGCAGCCATTCGACGT‐3′; ERK1–850‐S: 5′‐TACCTACAGTCT CTGCCCTCTAAA‐3′; ERK1‐CT‐AS: 5′‐AAGCGGGCTTCTCTTGGAAGAT‐3′; ERK1b‐NT‐S: 5′‐GTAAG CCGGCCACCAGC‐3′; and ERK1b‐CT‐AS: 5′‐CTGGGGGC‐AAAGACAGT‐3′. Total RNA was prepared using TRI reagent (Molecular Research Center Inc.) according to the manufacturer's instructions. For cloning of HA‐ERK1b and HA‐ERK1, the rat constructs obtained with RT‐PCR were ligated to the 3′ of HA and into the EcoRI and XhoI sites of pCDNA1 (Invitrogen).

### 2.6 Preparation of extracts from EJ tumor

EJ cells (1 × 10^6^ cells in 0.5 ml of PBS) were injected subcutaneously into 10 CD1‐Nude mice obtained from the Animal Breeding Unit of The Weizmann Institute of Science. Two weeks later, the mice were killed and the tumors, which were 1–2 cm in diameter, were removed and placed in PBS at 4°C. The tumors were transferred to ice‐cold buffer H (10 ml) homogenized by PCU (Kinetica; three pulses of 20 s) and disrupted by sonication (three pulses for 20 s at 50 W). The homogenate was centrifuged (100,000*g*, 30 min, 4°C) and the resulting supernatant was immediately applied to the anion exchange column at 4°C.

### 2.7 Anion exchange chromatography

Separations were performed using an AKTA system with a Resource Q column (20 ml; Amersham Pharmacia Biotech). After equilibrating with Buffer A, extracts (50 ml) were loaded at 1 ml/min. The bound proteins were then eluted (1 ml/min, 1 ml fractions) by increasing NaCl gradient (0%–30%). The flowthrough fractions containing ERK1b were collected and immediately applied to the affinity column at 4°C.

### 2.8 Affinity chromatography

The affinity column consisted of ERK1‐C16‐Ab‐agarose conjugate (0.75 ml; CS‐93‐AC, Santa Cruz Inc.). The flow‐through of Resource Q column was loaded (two times, 1 ml/min) onto the column, which was then washed once with 5 ml of 0.1 M glycine, 0.15 M NaCl, pH 2, and once with 0.1 M triethylamine, 0.15 M NaCl, pH 11. The column eluted with 4 ml (four fractions of 1 ml) of 0.1 M triethylamine, 0.15 M NaCl, pH 12.5.

### 2.9 Transfection of mammalian cells

COS7 cells, grown in 10 cm plates, were transfected using the diethylaminoethyl‐dextran method with 5 µg of HA‐tagged ERK1 (HA‐ERK1), HA‐ERK1b, or empty vector for immunoprecipitation and cotransfected with MEK1 together with either HA‐ERK1, HA‐ERK1b, or vector control (1:1 ratio, 5 µg total) for MEK1 coimmunoprecipitation. EJ, Rat1, and CHO cells, grown in 6 cm plates, were transfected or cotransfected using LipofectAMINE (Life Technologies, Inc.) with various plasmids (2.5 µg) as follows: HA‐tagged ERK1 (HA‐ERK1), HA‐ERK1b, MEK1, or empty vector.

### 2.10 Immunoprecipitation

One day after transfection, cells were serum starved (0.1% fetal calf serum) for an additional 16 h and then stimulated with either VOOH (100 µM Na_3_VO_4_ and 200 µM H_2_O_2_, 18 min, 37°C), epidermal growth factor (EGF; 50 ng/ml, Sigma), tetradecanoyl phorbol acetate (TPA, 250 ng/ml, Sigma), or PBS as control. After stimulation, the cells were washed, lysed, and the ERKs were incubated with a polyclonal anti‐HA‐Ab (Santa Cruz). For MEK1 coimmunoprecipitation studies, the beads were washed with low stringency buffer (20 mM HEPES, pH 8.0, 2 mM MgCl_2_ 2 mM EGTA) and then subjected to immunoblotting with monoclonal anti‐MEK and antigreen fluorescent protein Abs as previously described (Maik‐Rachline et al., 2018; Rubinfeld et al., 1999). For determination of ERK activity, the beads were washed once with 0.5 M LiCl, twice with radioimmune precipitation buffer, and once with buffer A as previously described (Silverman et al., 1999). The immunoprecipitates were subjected either to western blot analysis or to myelin basic protein (MBP) phosphorylation assay as described below.

### 2.11 Determination of ERK activity in vivo

Immunoprecipitates of ERK1 and ERK1b proteins were mixed with either MBP (8.4 µg), Elk1 (1 µg, NEB), or RSK (immunoprecipitated with RSK Abs (Sigma) from resting EJ cells and Buffer R that contained 100 µM [γ‐^32^P]ATP (1–2 cpm/fmol) in a final volume of 30 µl. The phosphorylation reaction was allowed to proceed at 30°C for 15 min and terminated by sample buffer followed by boiling for 5 min. Phosphorylated proteins were assessed by SDS‐PAGE and autoradiography or western blot analysis with DP‐ERK for phosphorylated ERKs. To separate between ERK1,2 and ERK1b, we used anion exchange column as previously described (Seger et al., 1994). Briefly, cell extracts (0.75 ml, 0.5 mg) were loaded on DE52 columns (0.4 ml) and the flow‐through fraction, containing ERK1b activity, was collected. After wash (1 ml × 3) with Buffer A + 0.02 M NaCl, the ERK1,2 activity were eluted with 1 ml of Buffer A + 0.22 M NaCl. The ERKs were then immunoprecipitated with ERK1‐Ab (C‐16) and subjected to in vitro phosphorylation as above.

### 2.12 In vitro activation of ERK

Extracts from nonstimulated transfected COS7 cells were subjected to immunoprecipitation with anti‐HA‐Ab. The HA‐ERKs proteins, attached to the beads, were mixed with DN‐EE‐MEK recombinant protein (Sigma) and Buffer R that contained 100 µM [γ‐^32^P]ATP (1–2 cpm/fmol) at 30°C. After 15 min, MBP (8.4 µg) was added (final volume of 30 µl) and the phosphorylation was allowed to continue for an additional 15 min. The reaction was terminated and assessed as described above.

### 2.13 Localization studies

CHO cells were transfected with either HA‐ERK1 or HA‐ERK1b together with MEK1. Immunoflouresence studies were performed as previously described (Jaaro et al., 1997). Briefly, 24 h after transfection, the cells were starved (0.1% fetal calf serum, 24 h) and stimulated (15 min, 37°C) with VOOH, fixed (3% paraformaldehyde), permeabilized (0.2% Triton X‐100, 5 min), washed with phosphate‐buffered saline, and stained with polyclonal anti‐HA‐Ab (diluted 1:100; Santa Cruz Biotechnology) and rhodamine‐conjugated goat‐antirabbit Ab (diluted 1:100; Jackson ImmunoResearch). Staining was visualized using a Zeiss fluorescent microscope.

## 3 RESULTS

### 3.1 Using anti‐DP‐ERK Abs to follow p46‐kDa ERK phosphorylation

**Figure 1:**
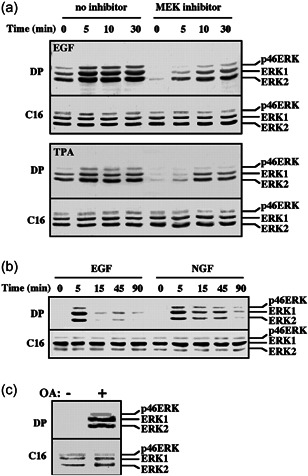
Stimulation of ERK1, ERK2, and p46 ERK phosphorylation in various cell lines. (a) Stimulation of extracellular signal‐regulated kinases (ERKs) phosphorylation in Rat1 cells. Rat1 cells were stimulated with epidermal growth factor (EGF; 50 ng/ml, upper panel) or tetradecanoyl phorbol acetate (TPA; 250 ng/ml, lower panel) for the indicated times in the absence (left side) or presence of the mitogen‐activated protein kinase/ERK kinase (MEK) inhibitor PD98059 (Biomol, 15 min prestimulation, 30 µM). (b) Extracts (containing cytosolic and nuclear proteins; 60 µg) of rat PC12 cells stimulated with EGF (50 ng/ml) or nerve growth factor (Sigma, 50 ng/ml) for the indicated times. (c) Extracts (50 µg) of human breast cancer‐derived MCF7 cells stimulated with okadaic acid (Sigma, 4 µM, 15 min, left) or left untreated (right). Each of these blots was immunoblotted with antibodies (Abs) to dually phosphorylated ERK (DP) and anti‐C terminus of ERK‐Ab (C16). The position of ERK2, ERK1, and p46 ERK is indicated. Each of these experiments was reproduced at least three times

The phosphorylation of ERK upon mitogenic stimulation of Rat1 cells was examined using the DP‐ERK Ab, which is specific to the dually phosphorylated TEY motif. To do so, we used extracts from serum‐starved and mitogen‐stimulated Rat1 cells, and subjected them to western blot analysis with the DP‐ERK Ab. We found that in addition to the expected 42‐ and 44‐kDa bands of ERK2 and ERK1, the Ab detected a faint band of 46 kDa (Figure 1a). Interestingly, the intensity of the band was increased upon stimulation with either EGF or TPA, with kinetics similar to those of the 42‐ and 44‐kDa ERK2 and ERK1. Although the Ab used are very specific, it was important to verify that the band recognize is of an ERK isoform and not a contaminant. For this purpose, we first used the specific MEK inhibitor PD98059. Thus, when this inhibitor was added before stimulation, the reactivity of the DP‐ERK Ab with all three bands was reduced. In addition, we used the antigenic peptide used to generate the Ab and found that it abolished the reactivity of the Ab with all three bands. Moreover, the 46‐kDa band was recognized by other anti‐ERK Abs, including some directed to the C or N termini of ERK1, Ab against subdomain XI of ERK1, and Ab against the C terminus of ERK2. We also found that this band is probably not related to other MAPKs at this molecular weight (MW), as anti‐p46 JNK1 did not recognize it. Thus, the band recognized by the anti‐DP‐ERK Ab seems to be a TEY‐containing ERK isoform, which is phosphorylated by MEKs. The band identified here might be the same one as a band with the same MW that was previously identified in rat (Boulton & Cobb, 1991; Peng et al., 1996) and possibly also in human (Gobert et al., 1995), which was tentatively termed ERK4.

The 46‐kDa ERK that we identified seems to be significantly phosphorylated in Rat1 cells. However, its expression or activation in other cell lines or tissues needed further consideration. For this purpose, we first examined several rat cell lines. Thus, when the rat‐derived PC12 cells were stimulated with either nerve growth factor or EGF, we observed the expected transient and sustained kinetics that was previously reported for ERK1 and ERK2 (Marshall, 1995), also for the 46‐kDa ERK (Figure 1b). We observed a sustained activation of the 46‐kDa ERK that was similar to the activation of ERK1, ERK2, also in FcγRI‐induced RBL‐2H3 cells (rat mucosal type mast cells), and in heat‐shocked glia cells from rat. These stimulations were intendent to achieve maximal ERK stimulation in these particular Rat‐derived cells. Interestingly, cells derived from other organisms had a much lower or no expression and phosphorylation of the 46‐kDa proteins. The increased phosphorylation upon stimulation was again similar to that of ERK1 and ERK2. This was seen in cells derived from mouse, calf, and guinea pig. Similar band, albeit with slightly higher MW (47 kDa), was seen in human breast cancer‐derived MCF‐7 cells in which a 47‐kDa ERK, ERK1, and ERK2 were significantly phosphorylated upon okadaic acid treatment (Figure 1c). However, later studies indicated that ERK1b is indeed found mainly rat cells and also in other rodents and calf, but not in primates (Aebersold et al., 2004). Therefore, the 47‐kDa ERK's bands seen in human or monkey are probably from a distinct origin, and do not represent the ERK1b isoform.

### 3.2 The 46‐kDa ERK in Ras‐transformed rat cells

**Figure 2:**
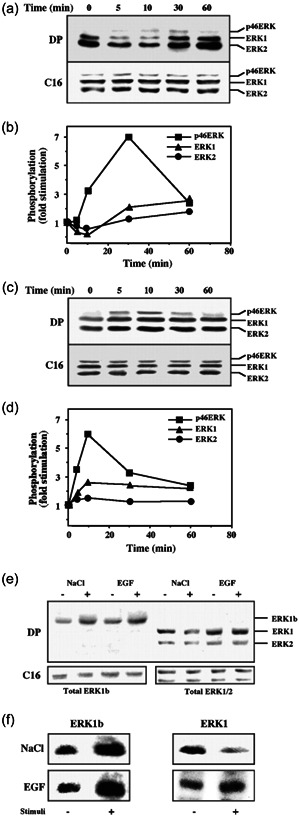
Kinetics of extracellular signal‐regulated kinase (ERK) phosphorylation and activation upon osmotic shock or epidermal growth factor (EGF) treatment of EJ cells. (a) Time course of ERK phosphorylation in NaCl stimulated EJ cells. EJ cells (7 × 10^5^ cells/6 cm plate) were serum‐starved for 16 h and then stimulated with 0.7 M NaCl, or with 50 ng/ml EGF. Extracts of cell from the indicated time points (50 µg) were analyzed by immunoblotting with doubly phosphorylated ERK (DP) and anti‐C terminus of ERK‐Ab (C16). (b) Quantitative determination of the results in (a). Phosphorylation of ERK2 (•), ERK1 (▲), and p46 ERK (■) was calculated as the intensity of staining with DP‐ERK divided by the intensity of staining with C16. This is a representative experiment that was reproduced four times. (c) Time course of ERK phosphorylation in EGF‐stimulated EJ cells. The cells were treated as in (a), except that the stimulation was with EGF (50 nM) for the indicated times. (d) Quantitative determination of the results in (c). Phosphorylation of ERK2 (•), ERK1 (▲), and p46 ERK (■) was calculated as the intensity of staining with DP‐ERK divided by the intensity of staining with C16. This is a representative experiment that was reproduced four times. (e) Determination of partially purified ERK1,2,1b phosphorylation. EJ cells were serum‐starved and then stimulated (+) for 10 min with either 0.7 M NaCl (NaCl), 50 ng/ml EGF (EGF), or left untreated (−). ERK1 and ERK2 (right four lanes) were separated from p46 ERK (left 4 lanes) on an anion exchange column, as described above. ERK activity in the different fractions was analyzed by immunoblotting with anti‐DP‐ERK and anti‐C terminus of ERK‐Ab (C16) as indicated. (f) Determination of partially purified ERK1,2,1b phosphorylation. ERK1/2 or ERK1b were prepared as described in (e) and their catalytic activity was determined by immunoprecipitation with ERK‐Ab (C16) followed by kinase assay with myelin basic protein (MBP) as a substrate, as described under Section 2. The results in (e) and (f) were reproduced four times

In all the above cell lines, the 46‐kDa ERK was significantly expressed and exhibited a similar kinetic of TEY phosphorylation as compared with that of ERK1 and ERK2. However, this was not the case in Ras‐transformed Rat1 cells (EJ), where the 46‐kDa ERK expression was higher than in nontransformed cells. Interestingly, such elevation in the 46‐kDa ERK expression upon Ras transformation was also shown in the rat PC12 (Robbins et al., 1992;  see Figure 1b) and rat IEC‐6 (Duhamel et al., 2012; see Figure 2a) cells, which indicates that this change in expression is a general phenomenon. Moreover, we found that in the EJ cells, phosphorylation of the 46‐kDa ERK varied significantly from that of ERK1 and ERK2. Thus, when these cells were osmotically shocked, the phosphorylation of ERK1 and ERK2 was reduced after 5–10 min and increased back to basal levels 60 min after. Staining with Abs to general ERK that recognize both phosphorylated and nonphosphorylated proteins (gERK Ab, C16 in this case) revealed no change in the total amount of ERK proteins (Figure 2a,b). This was not the case with the 46‐kDa ERK, whose phosphorylation was increased shortly after the osmotic shock, peaked at 30 min, and declined thereafter. Moreover, the kineticsof the 46‐kDa ERK phosphorylation was different when the same EJ cells were stimulated with EGF as well (Figure 2c,d). Similarly, the kinetics of the 46‐kDa ERK's phosphorylation was different from that of ERK1 and ERK2 also in mast cell function‐associated antigen (Rong & Pecht, 1996)‐stimulated mast cells. To further study this differential phosphorylation and confirm the distinct regulation of the 46‐kDa ERK, we partially purified these proteins using a small amount of DE‐52 resin (Seger, Seger, et al., 1992). We found that the 46‐kDa ERK was eluted from this anion exchange colon in the flowthrough, which was different from that of ERK1 and ERK2 that interacted with the DE‐52 and eluted with 0.2 M NaCl. Western blot analysis of the distinct fractions showed that, as expected from the result above, the flowthrough fractions also contained the 46‐kDa ERK that was detected with the DP‐ERK Ab. The intensity of the band was increased (five‐ to sixfold) after NaCl and EGF treatments, but the total amount of the 46‐kDa proteins was not changed (Figure 2E, left). The western blotting also showed that the fraction eluted at 0.2 M NaCl contained the 42‐ and 44‐kDa ERK2 and ERK1 but not the 46‐kDa ERK. Staining with the DP‐ERK Ab was reduced after osmotic shock and was not changed significantly when the cells were subjected to EGF stimulation. Again, staining with anti‐gERK Ab revealed no change in the total amount of ERK proteins in these fractions (Figure 2E, right). Next, we turned to follow the kinase activity of the ERKs and their activation. This was done using immunoprecipitation of the ERK proteins from each fraction with anti‐C‐terminal Abs, followed by an in vitro phosphorylation assay with MBP as a substrate. We found that the activity was directly correlated with the DP‐ERK Ab results in crude cell extracts that were shown above (Figure 2f). These results confirm that the 46‐kDa ERK is differentially regulated in EJ cells. Thus, although the mode of ERK1, ERK2, and the p46 kDa ERK regulation is usually similar, a differential mode of p46‐kDa ERK regulation may also exist. These distinct regulation may be derived from differences in dephosphorylation that is important for the activation under some conditions.

### 3.3 Cloning, purification, and Ab preparation of ERK1b

**Figure 3:**
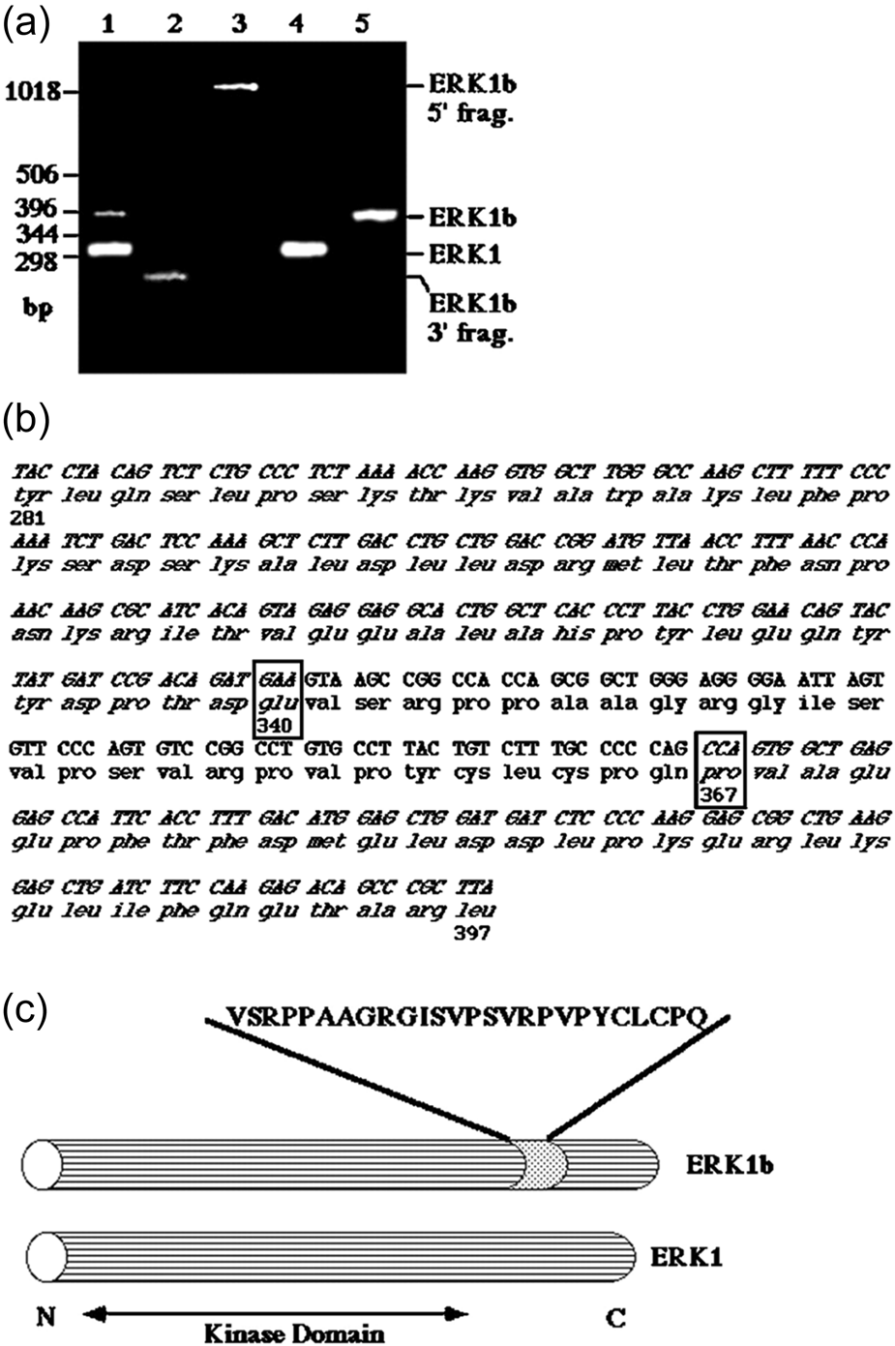
Cloning of ERK1b. (a) Reverse‐transcriptase polymerase chain reaction (RT‐PCR) and PCR cloning of ERK1b. Lane 1, RT‐PCR with the oligonucleotide primers ERK1–850‐S and ERK1‐CT‐AS, and total RNA from EJ cells as a template. Lane 2, RT‐PCR with the oligonucleotide primers ERK1b‐NT‐S, ERK1‐CT‐AS, and total RNA from EJ cells. Lane 3, RT‐PCR with the oligonucleotide primers ERK1b‐CT‐AS, ERK1‐NT‐S, and total RNA from EJ cells. Lane 4, PCR with ERK1–850‐S, ERK1‐CT‐AS, and ERK1 in pCDNA1. Lane 5, PCR with ERK1–850‐S, ERK1‐CT‐AS, and ERK1b cloned into the EcoRI and XhoI sites of pCDNA1. The position of the relevant fragments and DNA markers is indicated. (b) Complementary DNA (cDNA) and amino acid sequences of the ERK1b‐specific and some of the flanking sequence (italics). The insertion site (Glu‐340 and Pro‐367, instead of Pro‐341 in ERK1) in ERK1 is indicated by squares. (c) Schematic representation of ERK1b and ERK1. The sequence of the ERK1b‐specific insert and its kinase domain are indicated

The expression of the p46‐kDa ERK in a many cell types and the fact that it may be activated better than ERK1 and ERK2 in transformed cells led us to further study this protein. Due to the recognition by various anti‐ERK Abs with a distinct antigenic site we prepared oligonucleotide primers (sense and antisense) derived from the sequence of ERK1. These primers were then used in RT‐PCR using RNA template obtained from EJ cells, looking for distinct products produced by the same primers. Interestingly, most of the primer pairs gave just one product, but one of pairs produced two amplified products (Figure 3a). One of the products was the expected ERK1 ~300 bp but the other had ~400 bp. We then further cloned each of these products and sequenced them. As expected, the ~300 bp product had the ERK1 sequence, whereas the bigger product had 300 bp from ERK1, but additional 78 bp insert did not have any resemblance to rat ERK1, or any other known protein (Figure 3b). We then cloned the full‐length protein by using oligonucleotides from the unique 78 bp insert, as well as from the 5′‐ and 3′‐ends of rat ERK1. These oligonucleotides and RNA from EJ cells as a template were subjected to RT‐PCR and resulted in a full‐length complementary DNA (cDNA) of this ERK. This cDNA had the 78 bp inserted between bases 1020 and 1021 of ERK1, but all the rest of the sequence was identical to that of ERK1 (Figure 3c). Therefore, this result indicates that the messenger RNA (mRNA) is an alternative spliced form of ERK1, which we termed ERK1b. According to the results, the alternative splicing occurs in Intron 7, namely at the end of Exon 7 of the *ERK1* gene, which ends with the site of insertion (Pages et al., 1995). The 78 bp insertion encodes 26 amino acids (Figure 3c), which are localized between Glu‐340 and Pro‐341 of rat ERK1. This region is localized in the large loop L16 between the α helices αi and αL16, C‐terminally to the kinase domain core (Figure 3b). We then inspected the sequence for possible similarities with other proteins, but did not find any such homology within the data base. No protein motif or phosphorylation sites were found in this this sequence. This protein was also distinct from the putative ERK1psi that was previously reported (Boulton, Nye, et al., 1991). Thus, we clearly indicates the existence of an additional mRNA for ERK1b. This mRNA encodes for a putative protein kinase with a calculated mass of 45.6 kDa.

### 3.4 Purification of the ERK1b protein

**Figure 4:**
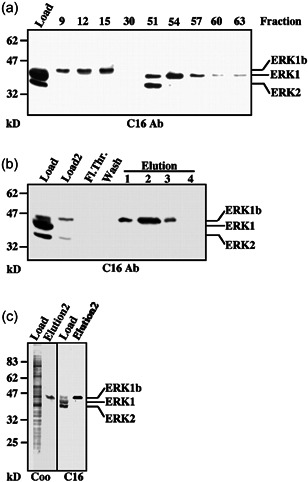
Purification of ERK1b. (a) Elution of a Resource Q column (Amersham Pharmacia Biotech) onto which the cytosolic and nuclear extract of EJ tumor cells (1 mg, Load) was loaded. After the flowthrough (1 ml fractions, 1–22), proteins were eluted by increasing NaCl gradient (0–0.3 M in 80 fractions, 1 ml/min, 1 ml fractions). The ERK content of the indicated fractions was examined by immunoblotting with anti‐C terminus of ERK‐Ab (C16). (b) The fractions containing ERK1b (fractions 8–17) from the Resource Q (Load2) were loaded on C16‐Ab affinity column. After washings (see “Section 2”), the ERK1b protein was eluted into four fractions (1.0 ml) with 0.1 M triethylamine containing 0.15 M NaCl, pH 12.5. The ERK content of the fractions was determined by immunoblotting with the C16‐Ab. (c) Analysis of the cytosolic extract and purified fraction (Elution2) with Coomassie Brilliant Blue staining (Coo) and immunoblotting with the C16‐Ab

To find out whether the 46‐kDa ERK is indeed expressed by the mRNA cloned, we needed to purify the endogenous protein and find out if it contains the expected sequence. For this purpose, tumors of EJ cells in nude mice were excised and then lysed in a buffer containing proteinase and phosphatase inhibitors. The extract was partially fractionated to cytoplasmic and nuclear fraction and the former was subjected to anion exchange column. As described above, unlike ERK1 and ERK2, the p46‐kDa ERK did not bind the resin and was eluted in the flowthrough (Figure 4a). The 46‐kDa ERK in that fraction was further purified using an affinity column consists of anti‐C terminus of ERK Ab bound to agarose. The column was then extensively washed and the bound proteins were eluted with high pH (Figure 4b). This procedure yielded ~4000‐fold purification of the 46‐kDa protein that was shown to react with various anti‐ERK Abs (Figure 4c) and was about 70% pure. We then further separated the p46‐kDa ERK using an SDS‐PAGE and the proteins between 40 and 50 kDa were excised and digested with trypsin. The resulting peptides were then analyzed by mass spectroscopy and when necessary also Edman degradation. We recovered eight peptides from the main protein in the excised band. Seven were from ERK1, including a peptide with the sequence around and including the TEY motif of ERK1. The eighth peptide corresponded to the sequence GISVPSVR, which is part of the predicted protein sequence of the 78 bp insert of ERK1b. This indicates that the isolated 46‐kDa band is indeed ERK1b.

**Figure 5:**
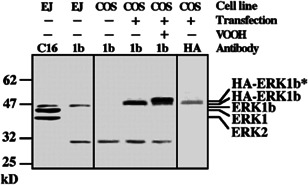
Preparation of anti‐ERK1b‐Ab. Ab to the 26‐amino acid insertion of ERK1b (Ab 4086) was developed as under “Section 2.” Cytosolic and nuclear extracts (50 µg) from nonstimulated EJ cells (EJ) or COS7 cells transfected with either pCDNA1 (−) or ERK1b (+) that were either stimulated with VOOH or left untreated were subjected to immunoblotting with anti‐C terminus of ERK‐Ab (C16), with Ab 4086 (1b), and with polyclonal anti‐HA‐Ab (hemagglutinin, HA). The experiment was reproduced three times

We then generated a polyclonal Ab directed to the specific insert of the 46‐kDa ERK1b. This Ab, which we termed 4086, detected two bands in a cytosolic extract of EJ cells (Figure 5); one of these bands was of 46 kDa, similar to a band recognized by anti‐C‐terminal ERK1. The other was 30 kDa, but this one was not competitively inhibited by the antigenic peptide, as did the 46‐kDa band, verifying the specificity of the Ab. To further confirm the specificity of the Ab, we overexpressed HA‐ERK1 and HA‐ERK1b in COS7 cells. Western blot analysis using Ab 4086 revealed that in mock‐transfected control no 46‐kDa protein detected by the Ab. However, as expected, this Ab recognized a 48‐kDa band in the HA‐ERK1b transfected cells. This band was also detected by an anti‐HA‐Ab and moved slower upon VOOH stimulation. No band was detected with HA‐ERK1. Thus, Ab 4086 specifically recognizes both endogenous and recombinant ERK1b, providing further evidence regarding ERK1b being the p46‐kDa ERK.

### 3.5 Functional characterization of ERK1b

**Figure 6:**
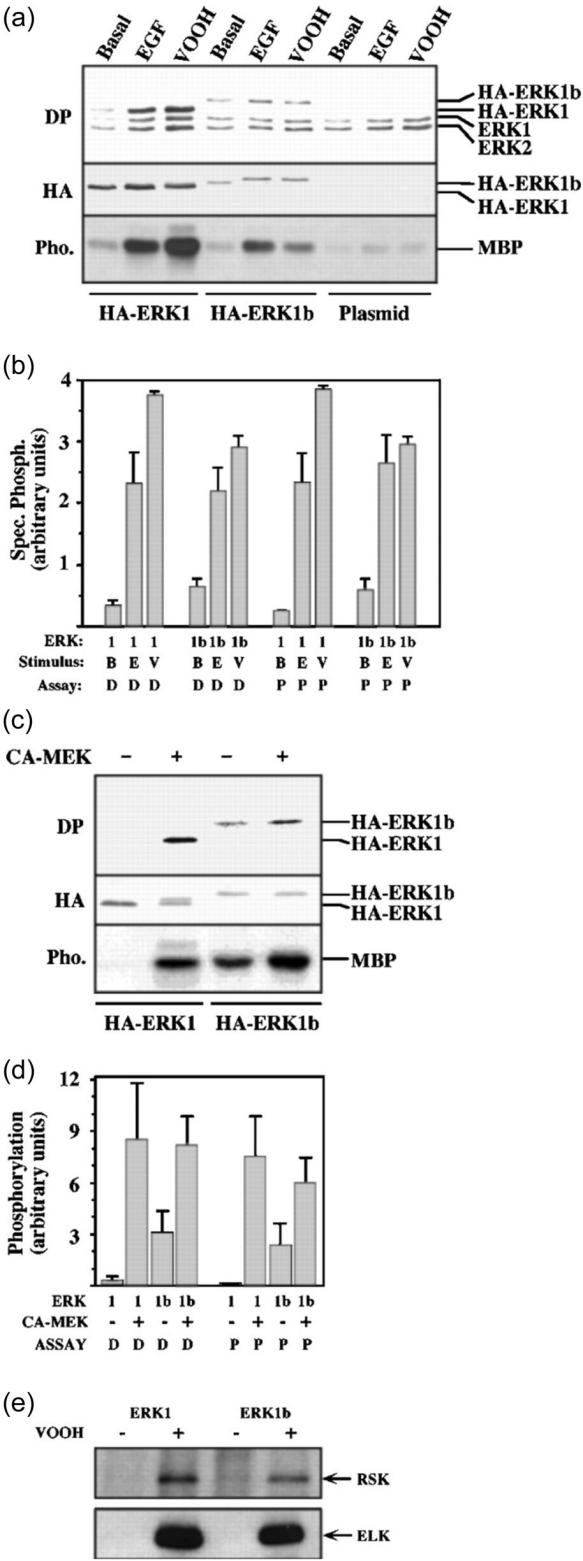
Expression and functional activity of ERK1b. COS7 cells were transfected with either HA‐ERK1b, HA‐ERK1, or pCDNA1 vector. (a) The cells were stimulated with either epidermal growth factor (EGF; 50 ng/ml, 10 min), VOOH (20 min), or left untreated (Basal). After harvesting, proteins were immunoprecipitated with polyclonal anti‐HA‐Ab and subjected either to western blot analysis with doubly phosphorylated extracellular signal‐regulated kinase (DP) and monoclonal anti‐HA‐Ab (hemagglutinin, HA) or to an in vitro phosphorylation of myelin basic protein (MBP) assay (Pho.). (b) The results in (a) were quantified and specific phosphorylation on the TEY motif (designated D) was calculated as the intensity of staining by DP‐ERK divided by the intensity of staining by HA‐Ab. Specific ERK activity toward MBP (designated P) was calculated as the amount of phosphorylated MBP minus the phosphorylation in the plasmid control per the intensity of staining by HA. Here, 1 stands for HA‐ERK1, 1b for HA‐ERK1b, B for Basal, E for EGF, and V for VOOH. (c) Immunoprecipitants from nonstimulated cells were subjected to an in vitro phosphorylation by constitutively active MEK (CA‐MEK; Jaaro et al.,^1997^) followed by either immunoblotting with DP‐ERK (DP) and a polyclonal anti‐HA‐Ab (HA) or MBP phosphorylation (Pho.) as in (a). (d) Specific phosphorylation of either the TEY sequence in ERK (DP) or in MBP was determined as in (b). The results in (b) and (d) are the average of two independent experiments. (e) Immunoprecipitants (with anti‐HA antibodies) from either nonstimulated cells (−) or VOOH‐treated cells (20 min, +) were subjected to in vitro kinase assay using either immunoprecipitated RSK (upper panel) or Elk1 (lower panel) as substrates. The amount of HA proteins in the examined fraction was similar (data not shown). The results in (e) were reproduced three times

Next, we were interested in the functional aspects of ERK1b, and whether it can operate similarly to the other ERKs. To do so, we transfected HA‐ERK1b, HA‐ERK1, and vector control to COS7 cells and then compared the activity of the kinases in extracts of serum‐starved, EGF, VOOH, or buffer control‐stimulated cells. As described above, this phosphorylation assay was done by immunoprecipitation of the proteins in these extracts, followed by an extensive washing to remove impurities. Next, the precipitated proteins were either subjected to SDS‐PAGE followed by western blotting with DP‐ERK and anti‐HA‐Ab, or subjected to a kinase assay using MBP as a substrate. Our results showed that the amount of expression of HA‐ERK1b was somewhat lower than that of ERK1 in COS7 cells when measured two days after transfection (Figure 6a). Interestingly, the specific activation (phosphorylation of the TEY motif per amount of HA‐ERK) or specific activity (phosphorylated MBP per amount of HA‐ERK) of HA‐ERK1b was found slightly higher than those of HA‐ERK1 (Figure 6b). However, stimulation slightly changes the situation, as ERK1b and ERK1 from stimulated cells (EGF and VOOH) had similar specific activation and specific activity. We then followed the ability of MEK to phosphorylate ERK1b in vitro. Thus, immunoprecipitated HA‐ERK1 and HA‐ERK1b were used as substrates for ΔN‐EE‐MEK1 (Jaaro et al., 1997). We found that ERK1 and ERK1b were slightly different in resting cells, but were potently activated, to similar levels, by active MEK, both reaching similar specific activity and specific activation (Figure 6c,d). As for the activity of ERK1b, we found that it is able to phosphorylate both Elk1 and RSK, almost to the same level as ERK1 in in vitro kinase assay (Figure 6e). Taken together, these results indicate that the 26 amino acids insert affects ERK1b in resting mammalian cells. However, it does not change its activation or its catalytic activity toward MBP, RSK, and Elk upon stimulation.

### 3.6 Tissue and organism distribution of ERK1b

**Figure 7:**
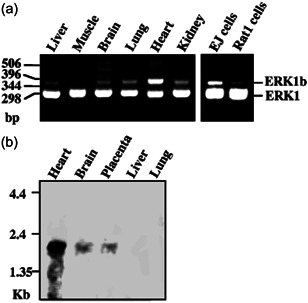
Tissues distribution of ERK1b. (a) Reverse‐transcriptase polymerase chain reaction (RT‐PCR) on rat total RNA from the indicated tissues and cells with ERK1–850‐S and ERK1‐CT‐AS primers. This RT‐PCRs was reproduced three times. (b) Northern blotting with the ERK1b 78 bp probe on the indicated tissues from human

It was previously shown that both ERK1 and ERK2 are expressed in all organisms and tissues, without a significant change under different conditions (Seger & Krebs, 1995). We then undertook to study the tissue distribution of ERK1b in rat by performing RT‐PCR on RNA from several rat tissues or cell lines. This was done using sense and antisense oligonucleotide primers, which are expected to yield a 303 bp product from ERK1 and a 381 bp product from ERK1b. Indeed, these two DNA products were seen in most tissues (Figure 7a). As expected, the amount of ERK1 product was roughly similar in the tissues and cell types sampled. On the other hand, ERK1b was expressed to a lesser extent in all tissues and its amount was varied in the distinct sources. The highest expression of ERK1b transcript was seen in the heart, whereas somewhat less ERK1b were also present in the kidney, lung, and brain. Finally, only a small ERK1b expression was detected in the liver and skeletal muscles. As for the EJ cells, in which the ERK1b protein is highly expressed (Figure 2a), the amount of ERK1b RNA expressed was much higher than that in Rat1 cells. We then tested human tissues using a ^32^P‐labeled probe that was prepared from the 78 bp insertion of the ERK1b cDNA. In northern blottings, the ERK1b probe identified a major 2.2 kb transcript mainly in the heart (Figure 7b), but also weakly in the brain and placenta. Thus, ERK1b RNA, although not restricted to rat, is not as ubiquitously expressed as the other ERKs.

### 3.7 ERK1b is differentially regulated in Ras‐transformed Rat1 cells

**Figure 8:**
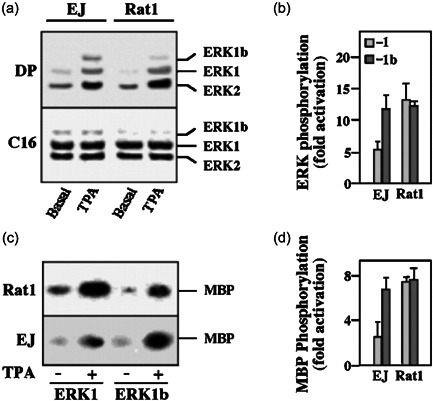
ERK1b is differentially regulated by tetradecanoyl phorbol acetate (TPA) in EJ cells. (a) EJ and Rat1 cells (~5 × 10^5^ cells/6 cm plate) were serum‐starved for 16 h and then stimulated (15 min) with TPA (250 nM) or left untreated (Basal). The cells were then harvested and 50 µg cytosolic and nuclear extract of each treatment were subjected to sodium dodecyl sulfate–polyacrylamide gel electrophoresis (SDS‐PAGE) and immunoblotting with either doubly phosphorylated extracellular signal‐regulated kinase (DP) or anti‐C terminus of ERK‐Ab (C16). The position of ERK1b, ERK1, and ERK2 is indicated. (b) Quantitative determination of fold stimulation of ERK1 (denoted 1) and ERK1b (denoted 1b). The intensity of the bands in A was determined by densitometer (model 690, Bio‐Rad). Specific phosphorylation of ERK1 and ERK1b was calculated as the intensity of staining with DP‐ERK of each of the band divided by the intensity of their counterpart band detected by the C16 antibody. Fold stimulation was calculated as the phosphorylation of TPA per basal phosphorylation and is the average of four independent experiments. Lightly shaded columns, ERK1b; dark shaded columns, ERK1. (c) Rat1 (upper panel) and EJ (lower panel) cells were transfected with either HA‐ERK1b or HA‐ERK1. After 36 h, the cells were serum‐starved for 16 h and then stimulated (15 min) with TPA (250 nM) or left untreated. Hemagglutinin (HA)‐containing proteins were immunoprecipitated with polyclonal anti‐HA‐Ab and subjected either to western blot analysis with monoclonal anti‐HA‐Ab (data not shown) or to in vitro phosphorylation of MBP. (d) Specific ERK activity was calculated as the amount of phosphorylated MBP after the treatment per the MBP phosphorylation in nonstimulated cells and is an average of two independent experiments. Lightly shaded columns, rat1 cells; dark shaded columns, EJ cells

As shown above, ERK1 and ERK1b are activated similarly in most cells, but not in the Ras‐transformed EJ cells, where the phosphorylation of ERK1b is stimulated in a different manner from that of ERK1 and ERK2 (Figure 2). To verify the distinct regulation, we compared ERK1b with that of ERK1 in the EJ and Rat1 cells. Thus, when we used anti‐C‐terminal Ab of ERK1, we found that the amount of ERK1b in EJ cells was significantly higher (six‐ to sevenfold) than in Rat1 cells, whereas the amount of ERK1 and ERK2 was roughly similar (C16; Figure 8a). Interestingly, elevated expression of ERK1b was noticed also with other oncogenes in other cell lines. Using the DP‐ERK Ab, we found that the basal activatory phosphorylation of all three ERKs ERK1, ERK2, and ERK1b was somewhat higher in EJ than Rat1 cells (Figure 8a). However, TPA stimulation caused a much higher phosphorylation of the ERK1b than the phosphorylation of ERK1 and ERK2 (Figure 8a,b). This was similar to the stimulation by EGF and NaCl (Figure 2). We further found that stimulation of the activatory ERK1‐TEY phosphorylation with TPA in EJ cells was moderate (up to sixfold; Figure 8b), significantly lower than the same stimulation in Rat1 cells (13.5‐fold). On the other hand, the stimulation of ERK1b‐TEY phosphorylation was higher in EJ cells as compared with the phosphorylation of ERK1 in Rat1 cells (12.5‐fold). To verify these results, we then overexpressed HA‐ERK1 and HA‐ERK1b in rat1 and EJ cells, and subjected the two to an in vitro kinase assay to measure ERK activity. As described above, this was done by immunoprecipitation with anti‐HA Abs and then in vitro phosphorylation of MBP. The results obtained with these methods (Figure 8c,d) was similar to that obtained with the anti‐DP‐ERK antibodies (Figure 8a,b). In particular, the activation of ERK1b with TPA was much higher (two to threefold) than that of ERK1 in EJ cells, but not in Rat1 cells. These results indicate that the regulation of ERK1b in EJ cells is different from the other ERKs, suggesting that ERK1b may be the major ERK isoform to respond to stimulation in transformed cells. Therefore, ERK1b may be important in regulating physiological responses in transformed cells.

### 3.8 The subcellular localization of ERK1b

**Figure 9:**
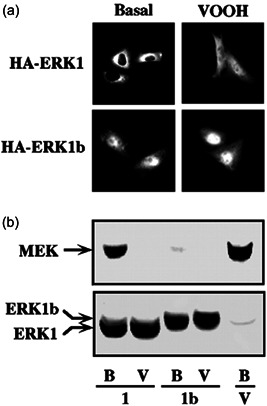
Mitogen‐activated protein kinase/extrecellular signal‐regulated kinase (MEK)‐dependent localization of ERK1 and ERK1b. (a) CHO cells were transfected with either hemagglutinin (HA)‐ERK1 or HA‐ERK1b together with wild‐type MEK1. Thirty‐six hours after transfection, the cells were serum‐starved for 18 h and then either stimulated with VOOH (100 µM Na_3_VO_4_ and 200 µM H_2_O_2_, 20 min) or left untreated (Basal). The ERKs were visualized by staining with polyclonal anti‐HA‐Ab and a fluorescent‐labeled secondary antibody (Ab) as described under “Section 2.” The amount of MEK was found to be similar in the cells examined (data not shown). This experiment was reproduced three times. (b) EJ cells were transfected with HA‐ERK1 (ERK1) or HA‐ERK1b (ERK1b) together with MEK1 (1:1 ratio of ERK containing plasmid to MEK containing plasmid) and treated as described above. After stimulation, the cells were collected, lysed, and the HA‐containing proteins were immunoprecipitated with polyclonal anti‐HA Ab, washed briefly (as described under “Section 2”), and subjected to western blot analysis with monoclonal anti‐MEK antibody (Ab; b, upper panel). The amount of ERK in the different lysates was determined by a western blot analysis with monoclonal anti‐HA Ab (b, bottom panel)

We then hypothesized that the differences in ERK1b regulation could derived from its distinct subcellular localization. Therefore, we followed the localization of ERK1b in CHO cells transfected with HA‐ERK1 or HA‐ERK1b. The cells were stained with anti‐HA‐Ab, revealing that most of the transfected proteins were localized in the nucleus before and after stimulation, without significant differences between them. These results are in agreement with our reported localization of overexpressed ERK in various cells (Rubinfeld et al., 1999). In those studies, we also showed that overexpressed MEK induces cytoplasmic retention of overexpressed ERK. Therefore, we coexpressed both HA‐ERK1 and HA‐ERK1b together with wild‐type MEK1 in CHO cells. As reported for ERK2 (Rubinfeld et al., 1999), we found that also HA‐ERK1 was localized primarily in the cytoplasm (90%) but translocated into the nucleus upon VOOH stimulation (Figure 9). Unlike this distribution, HA‐ERK1b was localized primarily in the nucleus (70%), even when coexpressed with MEK1, and slightly more so after stimulation (80%; Figure 9a). In view of these results, we assume that insert of ERK1b might be involved in disruption of the interaction with MEK and, therefore, MEK‐dependent cytoplasmic localization of ERK1b. This might be due to the effect of the insert on the conformation of the neighboring cytoplasmic localization sequence (residues 312–320 of ERK2) that we previously identified (Rubinfeld et al., 1999). To further verify the distinct MEK‐dependent localization of ERK1b, we then examined whether ERK1b interacts with wild‐type MEK. As expected (Rubinfeld et al., 1999), ERK1 interacted with MEK1 in resting cells and the interaction was reversed upon stimulation (Figure 9b). However, we found that ERK1b did not interact with MEK1 almost at all, even in resting cells (Figure 9b). These results support our hypothesis that the 26‐amino acid insertion indeed interferes with the MEK‐dependent cytosolic ERK1 anchoring and thus causes the nuclear localization of ERK1b. This differential localization seems to cause a different regulation of ERK1b by phosphatases and brings about the higher response to stimuli in transformed cells.

## 4 DISCUSSION

The ERK cascade is a central signaling pathway, which initiates and regulate variety of physiological processes to determine distinct cellular fates. Therefore, it is important to understand the mechanism of signaling specificity, namely the way that signals are transmitted by a given signaling cascade to induce different outcomes. Quite a few ways to determine signaling specificity have been proposed over the past years. These include the changes in strength and duration of signaling via the ERK cascade that was initially reported in PC12 cells (Shaul & Seger, 2007). In these cells, ERK transmit the signals to both proliferation and differentiation, where transient activation of ERK results in proliferation, while a sustained activation leads to differentiation. Other ways to dictate specificity were reported recently and those include compartmentalization that is evoked by interaction with cytoskeletal (Reszka et al., 1995) or scaffold proteins (Schaeffer et al., 1998). Another mechanism that may be involved is a cross‐talk between distinct signaling pathways, which may modify the signals in the ERK or other cascade (Sadot et al., 1998) or differential intensities of signals (Robinson & Cobb, 1997), which operate via additional signaling pathways. Finally, the signaling may be diverse because of multiplicity of differentially‐regulated isoforms in each layer of the cascade that are differentially activated by distinct stimuli. Here we demonstrate a way that may diversify the ERK cascade, in cloning and characterizing an alternatively spliced form of ERK1 (ERK1b), which operates differently than ERK1 (Figure 2), and therefore may contribute to signaling diversity and specificity.

Alternative splicing has already been reported to produce several isoforms of distinct proteins kinases and thereby modulate their downstream activities. In ERK, spliced isoforms at the mRNA level are ERK1psi (Boulton et al., 1990) and short ERK2 (Gonzalez et al., 1992), but it is not clear whether these proteins are significantly expressed in any cell or tissue. In addition, we identified an isoform of MEK1, termed MEK1b, which lacks 26 amino acids in subdomain V of MEK1, and is inactive towards ERK1/2 (Seger, Seger, et al., 1992; Zheng & Guan, 1993). Not less than 10 isoforms of JNK seem to be expressed in the human brain (Gupta et al., 1996), which were shown to differ mainly in their association with and regulation of the downstream targets ATF2, Elk‐1, and cJun transcription factors. These distinct associations lead to different effects of the JNKs in vivo. In addition, the upstream activator of JNK, MKK7, has no <6 spliced isoforms, and those differ in their association with JNK1, probably leading to distinct kinetics of activation (Tournier et al., 1999). Moreover, MEK5 acting in the ERK5 cascade also has several alternatively spliced isoforms and those seem to exhibit differential association with actin cytoskeleton (English et al., 1995). As a result, these isoforms may cause a differential subcellular localization of the two isoforms of MEK5. All the examples listed indicate that alternative splicing affects primarily protein‐protein interactions and localization of components of MAPK. The same effect is also observed for ERK1b and can account for its differential localization (Figure 9).

We show here that ERK1b is expressed mainly in rat cells, but some similarexpression was detected in human as well. This point led to a later work to explore the possible expression of ERK1b in other organisms. Using PCR, we found in a later study that insertion of Intron 7 to the mRNA indeed occurs also in human (Aebersold et al., 2004). Although the sequence of the human intron is not identical to the rat one, the sequence similarity is sufficient to detect its mRNA in some human tissues (Figure 7b). However, as this insert contains an in‐frame stop codon, its protein produced is smaller than the rat ERK1 and ERK1b, as well as the human ERK1, truncated just after the region that encodes the C‐terminal part of the kinase domain. This mRNA produces a 40‐kDa protein kinase that retains kinase activity. Moreover, due to its aberrant common docking (CD) domain (Rubinfeld et al.,^1999^; Tanoue et al., 2000) and lack of C‐terminal domain, the protein product has a distinct substrate specificity. Because of its difference from ERK1b, we termed this protein ERK1c and found it to be expressed in primates, particularly humans, but not in rodent or any other organisms (Maik‐Rachline et al., 2021). More studies on this human isoform revealed that it is activated by a distinct signaling route that includes MEK1b (Shaul et al., 2009). In addition, it was also shown that it translocates to the Golgi, where it regulates Golgi fragmentation during mitosis (Shaul & Seger, 2006; Wortzel et al., 2015). This effect is regulated by phosphorylating protein HOOK3 and possibly other Golgi proteins (Wortzel et al., 2021). These data indicate that alternatively spliced isoforms may significantly extend the specificity of the ERK cascade, but this may be different in distinct organisms.

Another point that is shown here is that the expression of ERK1b is amplified as compared with that of Rat1 in a MEK‐dependent manner (Figure 2a). This elevation, may be due to the oncogenic transformation of the cells. Indeed, oncogenic transformation was shown in the past to influence alternative splicing (Jücker et al., 1992). This effect of oncogenic transformation has not been studied yet, but might be due to the enhanced phosphorylation in the transformed cells. Indeed, phosphorylation is known to regulate alternative splicing (Wang & Manley, 1997) by affecting steps of the splicing processes (Misteli, 1999). Most notable are the SR proteins that are known to regulate the assembly of spliceosomes. In addition, these SR proteins may function as RNA splicing factors, whose activity is highly dependent on multiple phosphorylation (Kanopka et al., 1998). We found that in EJ cells, the expression of ERK1b is reduced by dominant negative MEK (data not shown). This suggests that phosphorylation by the ERK cascade is involved (directly or indirectly) in the regulation of alternative splicing upon malignant transformation. Another point is that in transformed cells such as EJ (Figure 8) or others (Salh et al., 1999), the constitutive activation by the driving oncogene upstream the ERK cascade is not always accompanied by a comparable activation of ERK. This may suggest that ERK1 and ERK2 are operating under a tight downregulation. Therefore, the fold stimulation of ERK1 and ERK2 by extracellular stimuli is low. On the other hand, ERK1b appears to escape this downregulation in the EJ cells and thus consists the major responsive ERK isoform (Figure 8).

We found here that the regulation of ERK1b localization is different from that of ERK1 and ERK2. When overexpressed in mammalian cells, ERK2 and ERK1 are found in the nucleus of resting cells. These changes in subcellular localization are seen mainly upon MEK1 co‐overexpression, which results mostly in cytosolic distribution of the ERK1 and ERK2 (Rubinfeld et al., 1999; Figure 9). On the other hand, ERK1b was not retained in the cytosol under similar conditions (Figure 9) and did not interact with MEK1. The reason might be an effect of the insertion on the binding to MEK. We recently showed that ERK‐MEK association is mediated by residues 312–320 of ERK2 and this region is responsible for MEK‐induced cytosolic retention of ERK (Rubinfeld et al., 1999). We showed that changing these residues to alanine residues led to a nuclear localization of ERK2, even in MEK1‐overexpressing cells. We termed this sequence the cytosolic retention sequence (CRS) and other named it CD motif (Tanoue et al., 2000). This CRS of ERK2 is almost identical to residues 332–340 of ERK1 and the ERK1b inserts lies just C‐terminal to it. Therefore, the insert likely changes the structure of the ERK1's CRS and causes a shift of ERK1b to the nucleus. Upon stimulation, when ERK1 and ERK2 transiently translocate into the nucleus, ERK1b stays in the nucleus under all conditions examined (Figure 9). Although the results were obtained using overexpressed ERK1b, they support our previous findings (Rubinfeld et al.,^1999^) on the importance of ERK's CRS in determining their subcellular localization.

Interestingly, the fact that the ERK1b is localized in the nucleus does not affect its activation upon mitogenic stimulation. Indeed, the kinetic of activation of this isoform was similar to that of the other ERKs under most condition. This result might be unexpected, as MEK is found primarily in the cytoplasm. Possible explanations is that MEK by itself translocate to the nucleus upon stimulation. Such an effect was found in the past, as we have shown that despite the usual cytosolic distribution of MEK1, this kinase does translocate into the nucleus upon mitogenic stimulation (Tolwinski et al., 1999). This nuclear translocation of MEK was suggested to play a role in the regulation of cell proliferation and oncogenesis (Fukuda et al., 1997), but the nuclear substrates for this MEK were not identified. It is possible that one of these substrates is the nuclear ERK1b, which serves as a substrate for the translocating MEK. Importantly, the stimulation of ERK1b was not always identical to those of ERK1 and ERK2. Few differences were observed in the kinetics of activation of this protein in Ras‐transformed EJ cells. These differences might occur by interaction of ERK1b with MAPK phosphatases (MKPs). These phosphatases are known to interact with Asp‐339 of ERK1 and this interaction was shown to be essential for their phosphatase activity (Camps et al., 1998). The 26‐amino acid insertion of ERK1b may change the mode of ERK1b‐MKP interaction to affect the regulation of the kinase. It is also possible that the regulation is mediated by other phosphatases that do not belong to the MKP family.

## 5 CONCLUSIONS

### 5.1 ERK1b is alternatively spliced isoform of ERK1 in rodents

We report here on the purification, cloning, and characterization of ERK1b. This is an alternatively spliced form of ERK1 and we show that it contains a 26‐amino acid insert located between residues 340 and 341 of ERK1. The activation by MEK and kinase activity towards MBP of ERK1b are very similar to those of ERK1. However, its tissue distribution is different, as ERK1b was detected mainly in rat, where it is abundant in the heart, as well as in a limited number of other organisms, but not primates.

### 5.2 Role in transformed cells

In addition, in Ras‐transformed Rat1 cells (EJ), the phosphorylation of ERK1b was differed from that of the other ERKs. Moreover, the localization of ERK1b was different from that of ERK1 and ERK2, as unlike the cytoplasmic distribution of the latter, ERK1b was found primarily in the nucleus. This difference might be due to change in the structure of CRS, which is located near the site of the ERK1b‐specific 26‐amino acid insert. This insert may also affect the association of ERK1b with MKPs.

### 5.3 Significance

Our findings are consistent with ERK1b being the major ERK isoform that responds to exogenous stimulation in transformed cells, at least in rodents.

## AUTHOR CONTRIBUTIONS

Yuval Yugn, Zhong Yao, and Tamar Hanoch performed the experiments Yuval Yung prepared the figures. Rony Seger supervised the work and wrote the article. All authors read and approved the final manuscript.

## CONFLICTS OF INTEREST

lThe authors declare no conflicts of interest.

## Data Availability

All data appear in this paper.
